# Necrotic bark of common pine (*Pinus sylvestris* L.) as a bioindicator of environmental quality

**DOI:** 10.1007/s11356-014-3355-0

**Published:** 2014-08-10

**Authors:** Anna Chrzan

**Affiliations:** Department of Ecology, Wildlife Research and Ecotourism, Institute of Biology, Pedagogical University of Krakow, ul. Podbrzezie 3, 31-084 Krakow, Poland

**Keywords:** Heavy metals, Necrotic pine bark, pH reaction, Niepołomice Forest

## Abstract

The aim of this study was to determine the pH and the concentration of lead, cadmium, nickel, copper and zinc in aqueous extracts of necrotic bark *Pinus sylvestris* L. and in adjacent soil, located in two types of forest habitat in different parts in the Niepołomice Forest in southern Poland. The Niepołomice Forest is located about 35 km east of an urban-industrial agglomeration Kraków. Despite the lack of significant differences in pine bark reaction studied, there was a clear difference in contamination of both bark and soil with heavy metals. There was a correlation between the distribution of pollutants in the forest, and the direction of the prevailing winds. More heavy metals were accumulated in the pine bark and soil from the west than the east. The high content of lead, zinc, cadmium and copper in the soils most likely results from the inflow of gas and dust pollutants from the urban-industrial agglomeration of Kraków.

## Introduction

Biosphere pollution imposes a need to search for bioindicators enabling evaluation of the quality of environment and identification of trends in its changes. Using biological materials in the determination of environmental pollution as indicators is a cheap and reliable method (Faggi et al. 2011). Various types of plant, lichens, mosses, bark and leaves of higher plants have been used to detect the deposition, accumulation and distribution of metal pollution (Grodzińska 1982; Sawidis et al. 2011; Serbula et al. 2013). Tree bark is a sensitive indicator of environmental pollution, particularly of acidifying compounds and heavy metals (Al-Asheh and Duvnjak 1997). These contaminants affect the physicochemical properties of bark (Świeboda and Kalemba 1979; Santamaria and Martin 1997). The pH depends on the species, age and the health of the trees and on the soil they grow on (Medwecka-Kornaś et al. 1989; Szczepanowicz and Gawroński 2000). Among the studied barks of pine tree are Turkish red pine *Pinus brutia* Ten. (Baslar et al. 2009), Italian stone pine *Pinus pinea* L. (Oliva and Mingorance 2006), Austrian pine *Pinus nigra* Arnold. (Coskun 2006), Masson pine *Pinus massoniana* Lamb. (Kuang et al. 2007) and Scots pine (*Pinus sylvestris* L.) (Świeboda and Kalemba 1979; Huhn et al. 1995; Lippo et al. 1995; Poikolainen 1997; Schulz et al. 1999; Pöykiö et al. 2005; Saarela et al. 2005; Samecka-Cymerman et al. 2006). In Poland, atmospheric pollution may be well detected with the bark of common pine (*P. sylvestris* L.), being a sensitive and common (over 70 % of all Polish trees) bioindicator, located at fixed sites, constantly influenced by the surrounding environment and easily sampled and analysed (Grodzińska 1978, 1982; Marko-Worłowska et al. 2011; Chrzan and Marko-Worłowska 2012). Examination of water extracts from tree bark provides data on its pH reaction (lowered by SO_2_ and NO_*x*_ in the air), electrolytic conductivity, ability to absorb chosen chemical elements and concentration of sulphates in extract (Schulz et al. 1999).

Soil also belongs among the components of the natural environment more sensitive to the effects of pollution, including pH changes caused by human activity (Kowalkowski 2002; Hernandez et al. 2003). Acidity is one of the key factors determining the course of many soil processes, affecting the functioning and efficiency of entire geoecosystems. Basically, it affects the living conditions of soil organisms, the availability of macro- and micronutrients necessary for plant growth and the processes of nitrification and the presence of toxic heavy metals (Gambuś and Gorlach 2001; Gruca-Królikowska and Wacławek 2006).

The study aimed to determine the pH reaction and concentration of lead, cadmium, nickel, copper and zinc in water extracts from necrotic bark of P. *sylvestris L.* and in soil surrounding the analysed trees, situated in two forest habitat types of the western and southern part of the Niepołomice Forest located in southern Poland, as well as to evaluate the environmental pollution in this area on the basis of abovementioned parameters.

## Materials and methods

The study area covered the Niepołomice Forest, located ca. 35 km from Kraków and since many years considered as its “green lungs”. The area extends at ca. 10,758 ha, includes ca. 80 % of forest surface in the Niepołomice Forest District and includes several parts, presently separated, however once integrated.

**Fig. 1:**
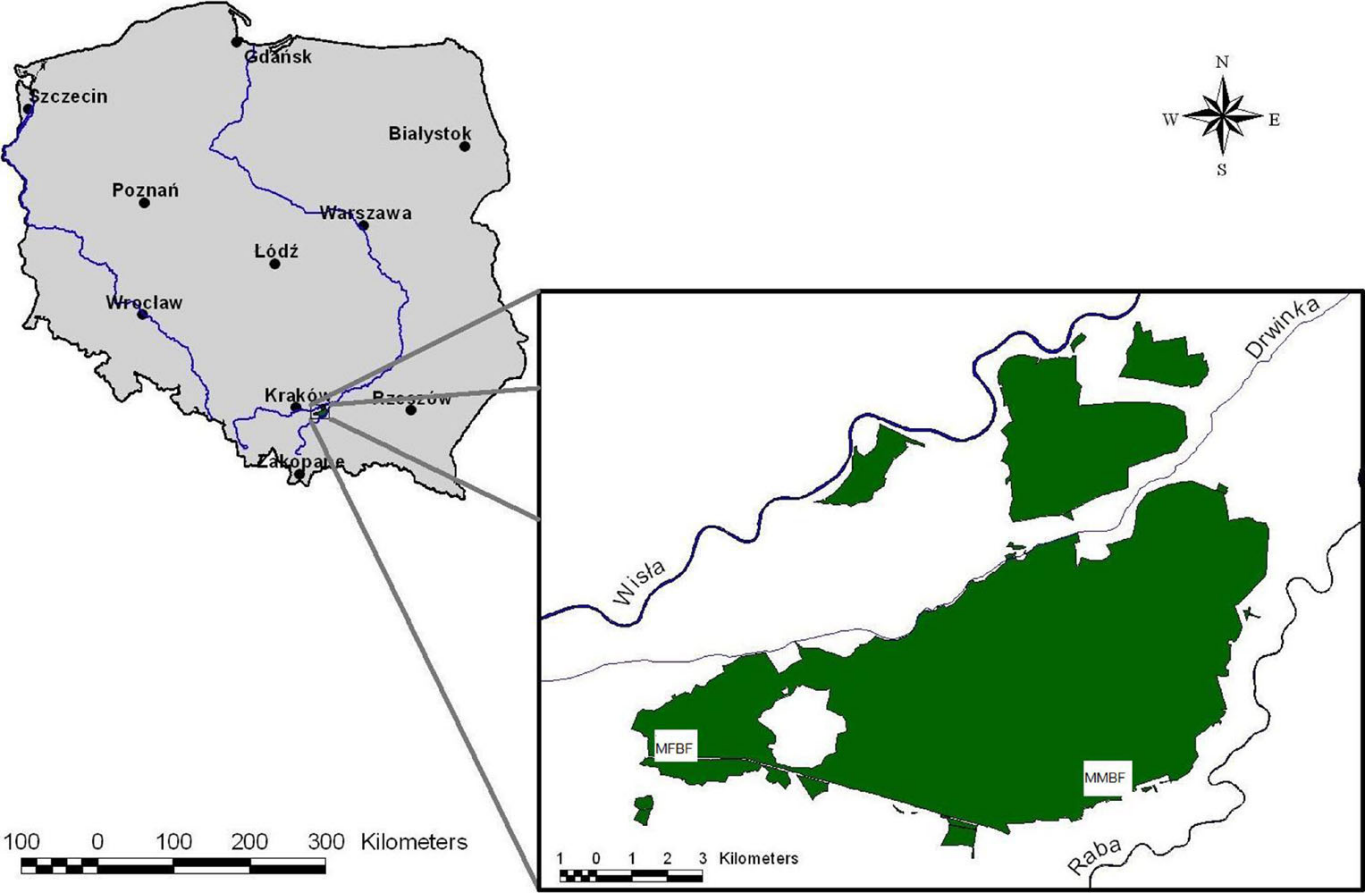
Place of research in the Niepołomice Forest

Species composition in stands of the Niepołomice Forest is dominated by pine, attaining a frequency of 62 %. Bark was sampled from even-aged specimens of common pine growing in two sectors of the Niepołomice Forest, namely the fresh mixed broadleaved forest (in the western part of the southern complex of the Niepołomice Forest, in the Sitowiec Forestry), and the moist mixed broadleaved forest (in the ecotone zone of the southern part of the Niepołomice Forest, in the Baczków Forest District, near a highway, built at that time) (Fig. 1). The areas were covered by soils of similar type, namely brown acidic soils and podzols. All the samples were collected in the autumn in October.

Both sectors included trees of similar size (diameter at breast height of ca. 40–45 cm) and age (ca. 45 years) and spaced at ca. 3–5 m from each other. For each tree, three samples of necrotic bark were collected, using a sharp knife, from all four sides of the trunk (southern, northern, western and eastern), at ca. 1.5 m from the ground. Surfaces covered with lichens or resin was avoided. Sampling was performed after a no-rainfall period, as recommended by other authors using the “bark test” (Medwecka-Kornaś et al. 1989). Additionally, topsoil samples, each of ca. 10 dag, were collected with a soil samples from the surrounding of examined trees, from the depth of ca. 5 cm. In total, 96 samples of pine necrotic bark (P. *sylvestris* L.) and 24 soil samples were taken.

The obtained bark was dried in open air at ca. 25 °C, for ca. 30 days, and ground to powder in an electric grinder. Samples were prepared by mixing ca. 1 g of the powdered bark with 8 ml of distilled water, at room temperature, and after 24 h were subjected to pH measurements using a WTW 330 pH meter. Similarly, all soil samples were dried and used to prepare solutions, including 10 g of soil and 25 ml of distilled water, in which pH was measured after 24 h from pouring water on to the soil. The next step of analytical studies involved preparation of soil and bark for mineralization. From each sample, 0.5 g of bark or ca. 1 g of soil was weighted out into a vial, afterwards filled with 4 ml of 65 % nitric acid. After mineralization (2–3 h at 110 °C) solutions were poured to volumetric flasks and filled with distilled water to 10 ml. Content of heavy metals (lead, cadmium, copper and zinc) in the mineralized soil and bark samples was determined in the AAS atomic absorption spectrophotometer (Cole-Parmer, BUCK 200A).

All the analyses were performed using STATISTICA 10 computer program. Results were subjected to analysis of variance with repeated measurements and Tukey’s test. Relationships between the pH reaction in bark and soil and metal content in bark and soil were assessed with the Pearson linear correlation coefficient. The *p* value assumed as statistically significant was *p* < 0.05 and statistically highly significant was *p* < 0.01.

## Results and discussion

The pH of pine bark P. *sylvestris* L. indicated its much higher acidity than that of the soil in which these pine trees grew. The pH of the analysed pine bark ranged from pH 2.92 to pH 3.25 (Table 1). Similar acidity of pine bark (2.97–3.16) showed Bąbelewska (2013) in forest reserves, “Zielona Góra” and “Sokole Góry” near Częstochowa in Poland. Santamaria and Martin (1997) also recorded similar bark acidity for P. *sylvestris* L (3.17) in Navarra, Spain. A little lower pH values of the pine bark obtained in a study in the Niepołomice Forest by Grodzińska (1978, 1982). The Niepołomice Forest, located just a few kilometers from the large agglomeration of Krakow and a steel mill, reported acidification of pine bark in the range 2.2–3.0 (Grodzińska 1982). Grodzińska (1978) reported similar values of pH reactions in pine barks of Kraków and polluted areas of southern Sweden.

**Table 1:** Comparison of pine bark and soil (descriptive statistics)

Parameters	X	SD	Min	Q25	Me	Q75	Max
pH bark W	3.10	0.05	3.02	3.06	3.08	3.12	3.21
pH bark E	3.13	0.08	2.99	3.06	3.12	3.19	3.25
pH bark N	2.92	0.17	2.66	2.83	2.87	3.05	3.25
pH bark S	2.76	0.09	2.63	2.70	2.74	2.79	2.97
pH soil W	4.66	0.68	4.11	4.20	4.38	4.98	6.35
pH soil S	4.14	0.17	3.89	4.01	4.10	4.27	4.45

*X* mean, *SD* standard deviation, *Min* minimum, *Q25* lower quartile, *Me* median, *Q75* upper quartile, *Max* maximum, *W* West, *E* East, *N* North, *S* South

The low pH of pine bark was also reported in Ojcowski National Park (2.6–3.5) (Medwecka-Kornaś et al. 1989), the most polluted national park in Poland.

Lowering the pH of pine bark below 3.0 is dangerous and can result in changes in the health of the forest ecosystem. pH of common pine bark lower than 3.0 may increase the accessibility of heavy metals to plant tissues and therefore threaten the healthiness of forest ecosystems (Bąbelewska 2013).

Differences between the pH reaction of bark collected from various sides of the examined pine trunks appeared to be highly significant (*F* = 31.49; *p* < 0.0001), with the highest values recorded for the western and eastern side (mean pH 3.1). These two groups did not differ statistically from each other (*p* = 0.9027), however showed significantly higher values than bark pH at the northern side (mean 2.92). The lowest bark pH was determined for the southern side (mean 2.76), statistically different from pH at W (*p* = 0.0002), E (*p* = 0.0002) and N (*p* = 0.003).

**Fig. 2:**
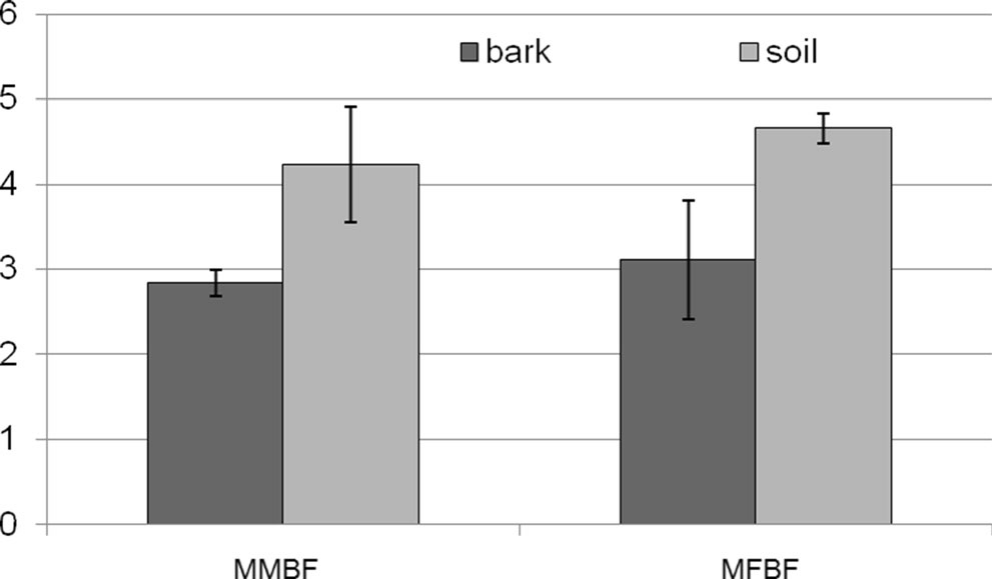
Comparison of the soil pH and pine bark in the studied types of forest habitat *MMBF* moist mixed broadleaved forest, *MFBF* fresh mixed broadleaved forest

Soil pH was generally higher, with values ranging between 3.9 and 6.35 and attaining a mean of ca. 4.5 (Table 1). The investigated forest habitat types showed only small differences in soil and bark pH, being slightly higher in the fresh than in the moist forest (Fig. 2). However, no statistically significant relationship was established between bark and soil pH at any of examined sides (*p* = 0.5174; *p* = 0.7253).

Concentration of metals in bark varied at particular sides of collection and was also different from soil. At the western side, necrotic bark of the examined trees displayed the highest heavy metal concentrations, with Pb accumulated at greatest values (42.35 mg kg^−1^ d.m.), followed by Zn (40.85 mg kg^−1^ d.m.), Cu (25.63 mg kg^−1^ d.m.) and Cd (2.08 mg kg^−1^ d.m.). Such high metal accumulation in the west-side bark may result from the direction of air movement in the Niepołomice Forest, dominated by western winds, carrying dust from the industrial agglomeration of Kraków. In contrast, the lowest metal content was generally recorded for bark from the southern side of trees (Zn—12.92 mg kg^−1^ d.m., Pb—9.63 mg kg^−1^ d.m., Cu—4.74 mg kg^−1^ d.m.).

In areas less intensively affected by anthropogenic activity, accumulation of Zn is lower than in industrial areas and roadsides (Baslar et al. 2009).

The minimum mean zinc value was determined as 8.4 mg kg^−1^ d.m. in control areas, and the maximum mean value was determined as 14.1 mg kg^−1^ d.m. in industrial areas and roadsides in Western Anatolian part of Turkey (Baslar et al. 2009). Higher concentrations of Zn (up to 189 mg kg^−1^ d.m.) showed by Schulz et al. (1999) in Rösa, a polluted area in Germany.

**Fig. 3:**
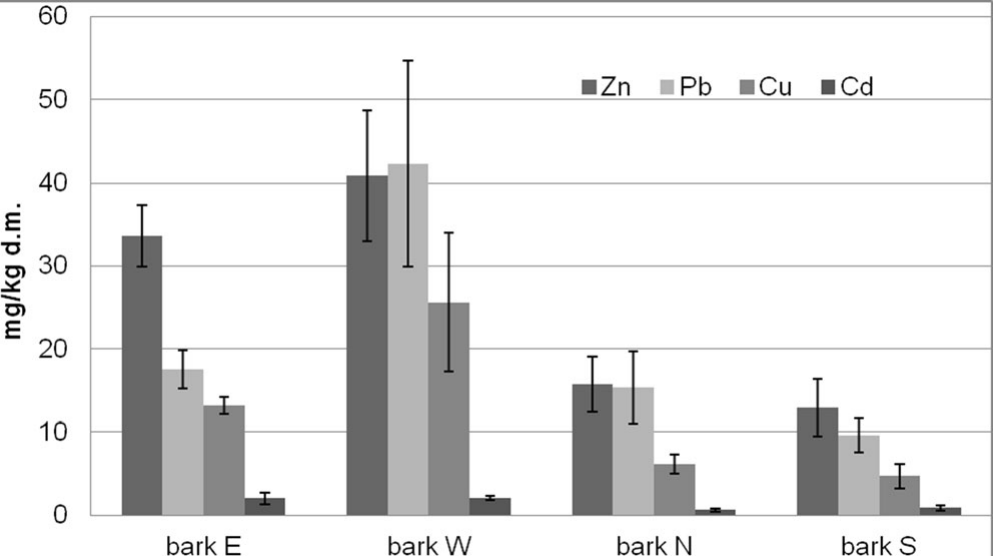
Content of heavy metal in pine bark milligram per kilogram d.m. *W* West, *E* East, *N* North, *S* South

Cadmium concentration in bark was the lowest at the northern side (0.62 mg kg^−1^ d.m.), while at the southern side, it showed a significant (*p* = 0.0403) and negative (*r* = −0.9456) correlation with Cd concentration in soil (Fig. 3). Schulz et al. (1999) showed in their work significantly lower mean of the metal—three times lower in Jostedalsbreen National Park in Norway, Syktyvkar in Russia and Neuglobsow in Germany, twice lower in Rösa, a polluted area in Germany, and only 1.5 times lower (0.4 mg kg^−1^ d.m.) in Białowieża, Poland.

**Fig. 4:**
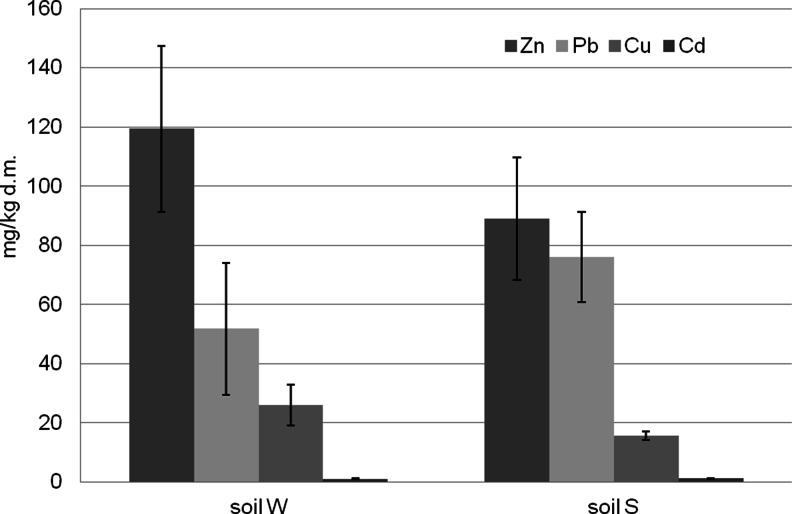
Content of heavy metal in topsoil milligram per kilogram d.m. *W* West, *S* South

Amounts of Zn, Cu and Pb attained much higher values in soil than in bark (Figs. 3 and 4).

Compared to bark, mean Zn content in soil was over six times greater in the southern part of the forest and three times greater (119.33 mg kg^−1^ dry weight) in the western part (Fig. 3). However, correlations between soil and bark for Zn concentration were not statistically significant (*p* > 0.05). A significant positive correlation was observed for Cu content in soil at the western side and in bark at the eastern side (*p* = 0.0422, *r* = 0.9404).

Soils, specifically their humic horizon, are the main site of accumulation and strong fixation of anthropogenically derived lead. Soil Pb contamination is rather the result of many years of deposition enriched in transboundary leaded gasoline particles than of a local or regional industrial sources (Hernandez et al. 2003). The element, though of low mobility, in acidic and sandy grounds may become easily accessible to plants and consequently can be incorporated into food chains and impose a direct threat to organisms (Kabata-Pendias and Pendias 1999). In Poland, lead concentration in soil attains a mean value of 18 mg kg^−1^ and is most frequently recorded at amounts not exceeding 100 mg kg^−1^. Soil of the examined fresh mixed broadleaved forest included 52 mg kg^−1^ Pb (Table 2). However, Pb from airborne particles represents the present-day atmospheric conditions, whereas the soil surface reflects several years of atmospheric deposition.

**Table 2:** The average of heavy metal concentrations (mg kg^−1^ d.m.) in soil and pine bark in Niepołomice Forest

Metal	MMBF soil	MFBF soil	MMBF bark	MFBF bark
Zn	89.44 (20.81)	119.33 (87.98)	14.33 (3.13)	37.23 (6.54)
Cu	15.86 (1.55)	26.00 (16.9)	5.46 (1.31)	19.39 (8.19)
Cd	0.93 (0.16)	0.88 (0.69)	0.76 (0.24)	2.06 (0.42)
Pb	76.11 (25.35)	51.76 (22.28)	12.48 (4.08)	29.93 (14.98)

Numbers in the bracket are the standard deviations of the concentrations (SD)

*MMBF* moist mixed broadleaved forest, *MFBF* fresh mixed broadleaved forest

This value was significantly and positively correlated with Pb content in bark (*p* = 0.0449, *r* = 0.9404). However, no correlation between concentration in soil and bark was established for other metals (*p* > 0.05).

The obtained results indicate increased content of copper, zinc, lead and cadmium in the investigated soils of the Niepołomice Forest. Much lower concentration of Pb (average 12.45 mg kg^−1^ d.m.), Cu (10.44 mg kg^−1^ d.m.) and Zn (39.5 mg kg^−1^ d.m.) were showed in a natural reserve in Dinghushan in China (Kuang et al. 2007). The obtained results are in consent with studies by Hernandez et al. (2003), who concluded that high metal content in soils may be recorded both close to pollution sources and at greater distances, what results from the geographic location of sites and spreading of pollutants by winds, in the direction of their movement.

Enrichment of forest topsoil with heavy metals is likely to evidence their anthropogenic origin. Such a quality of soil environment results possibly from the inflow of gas and dust pollutants carried by western winds, mainly from the urban-industrial agglomeration of Kraków. Consequently, soils of the western part of the Niepołomice Forest accumulated more zinc and copper, while soils of the southern part of the forest, adjacent to a highway, accumulated more cadmium and lead (Fig. 4).

Research of other authors (Härtel and Grill 1972; Poikolainen 1997; Harju et al. 2002; Kuang et al. 2007; Kakulu 2003; Sawidis et al. 2011; Guéguen et al. 2012) has confirmed that industrial activity tends to increase the concentration of metallic contaminants of barks. According to Huhn et al. (1995) and Schulz et al. (1999), heavy metal concentrations of the barks were generally found to correlate very well with the heavy metals deposition.

The results of researches from Niepołomice indicate that concentrations of analysed heavy metals do not exceed boundary values defined in the Regulation of the Minister of Environment on soil quality standards and earth quality standards for group B soils, including the forest, tree-covered and shrub-covered soils, wastelands and built-up and urbanized areas, however excluding industrial areas, mining grounds and transport areas (Regulation 2002). Similar results were reported by other authors (Cieśla et al. 1994; Skwaryło- Bednarz 2006).

## Conclusions

Tree bark is a sensitive indicator of environmental pollution, particularly of heavy metals. The presented results confirm the usefulness of water extracts from bark of common pine (*P. sylvestris* L.) in research on the spatial variation and distribution of pollution in forests. Bark of trees examined in the western part of the Niepołomice Forest showed higher concentrations of Pb, Cd, Zn and Cu than in the southern part, most likely due to accumulation of pollutants inflowing from the urban-industrial agglomeration of Kraków with the dominant western winds.

## References

[CR1] Al-Asheh S, Duvnjak Z (1997). Sorption of cadmium and other heavy metals by pine bark. J Hazard Mater.

[CR2] Bąbelewska A (2013). The impact of sulphur dioxide from Częstochowa Agglomeration on acidity degree of *Pinus sylvestris* L. bark of „Zielona Góra” and „Sokole Góry” Nature Reserves (Wyżyna Krakowsko-Częstochowska). Natural Environment Monitoring.

[CR3] Baslar S, Dogan Y, Durkan N, Bag H (2009). Biomonitoring of zinc and manganese in bark of Turkish red pine of western Anatolia. J Environ Biol.

[CR4] Chrzan A, Marko-Worłowska M (2012). Content of heavy metals in soil and in pine bark in Skalki Twardowskiego Landscape Park in Krakow. Ecol Chem Eng A.

[CR5] Cieśla W, Dąbkowska-Naskręt H, Borowska K, Malczyk P, Długosz J, Jaworska H, Kędzia W, Zalewski W (1994). Trace elements in soils of selected areas Pomorza and Kujaw. Zesz Probl Postep Nauk Rol.

[CR6] Coskun M (2006). Toxic metals in the Austrian pine (*Pinus nigra*) bark in the Thrace region, Turkey. Environ Monit Assess.

[CR7] Faggi AM, Fujiwara F, Anido C, Perelman PE (2011). Use of tree bark for comparing environmental pollution in different sites from Buenos Aires and Montevideo. Environ Monit Assess.

[CR8] Gambuś F, Gorlach E (2001). Origin and harmful heavy metals. Aura.

[CR9] Grodzińska K (1978). Acidity of tree bark as bioindicator of forest pollution in Southern Poland. Water Air Soil Pollut.

[CR10] Grodzińska K (1982) Monitoring of air pollutants by mosses and tree bark. In: Steubing L, Jager HJ (eds) Monitoring of air pollutants by plants: methods and problems. Proc Intern Workshop (1981). Junk, The Hague Boston London, 33–42

[CR11] Gruca-Królikowska S, Wacławek W (2006). Metals in the environment. Part II. Effect of heavy metals on plants. Chem Didact Ecol Metrol.

[CR12] Guéguen F, Stille P, Lahd Geagea M, Boutin R (2012). Atmospheric pollution in an urban environment by tree bark biomonitoring – Part I: trace element analysis. Chemosphere.

[CR13] Harju L, Saarela K-E, Rajander J, Lill J-O, Lindroos A, Heselius S-J (2002). Environmental monitoring of trace elements in bark of Scots pine by thick-target PIXE. Nucl Instrum Methods Phys Res B.

[CR14] Härtel O, Grill D (1972). Die Leitfahigkeit von Fichtenborken – Extrakten als empfindlicher Indikakor für Lufverunreinigungen. Eur J For Res.

[CR15] Hernandez L, Probst A, Probst JL, Ulrich E (2003). Heavy metal distribution in some French forest soils: evidence for atmospheric contamination. Sci Total Environ.

[CR16] Huhn G, Schulz H, Stark HJ, Tolle R, Schurmann G (1995). Evaluation of regional heavy metal deposition by multivariate analysis of element contents in pine tree barks. Water Air Soil Pollut.

[CR17] Kabata-Pendias A, Pendias H (1999) Biogeochemistry of trace elements. Państwowe Wydawnictwo Naukowe, Warszawa. (in Polish)

[CR18] Kakulu SE (2003). Trace metal concentration in roadside surface soil and tree barks: a measurement of local atmospheric pollution in Abuja, Nigeria. Environ Monit Assess.

[CR19] Kowalkowski A (2002). Ecochemical indicators of forest soil condition damaged by acidification. Natural Environment.

[CR20] Kuang YW, Zhou GY, Wen DZ, Liu SZ (2007). Heavy metals in bark of *Pinus massoniana* (Lamb.) as an indicator of atmospheric deposition near a smeltery at Qujiang, China. Environ Sci Pollut Res.

[CR21] Lippo H, Poikolainen J, Kubin E (1995). The use of moss, lichen and pine bark in the nationwide monitoring of atmospheric heavy metal deposition in Finland. Water Air Soil Pollut.

[CR22] Marko-Worłowska M, Chrzan A, Łaciak T (2011). Scots pine bark, topsoil and pedofauna as indicators of transport pollutions in terrestrial ecosystems. J Environ Sci Health Part A.

[CR23] Medwecka-Kornaś A, Kozłowska H, Gawroński S, Matysiak E (1989). The properties of extracts of the pine bark (*Pinus sylvestris* L.) as a pollution indicator in the Ojcowski National Park. Fragm Florist Geobot Polon.

[CR24] Oliva SR, Mingorance MD (2006). Assessment of airborne heavy metal pollution by aboveground plant parts. Chemosphere.

[CR25] Poikolainen J (1997). Sulphur and heavy metal concentrations in Scots pine bark in northern Finland and the Kola Peninsula. Water Air Soil Pollut.

[CR26] Pöykiö R, Perämäki P, Niemelä M (2005). The use of Scots pine (*Pinus sylvestris* L.) bark as a bioindicator for environmental pollution monitoring along two industrial gradients in the Kemi–Tornio area, northern Finland. Int J Environ Anal Chem.

[CR27] Regulation of the Minister of the Environment (2002) on the standards of the soil quality and ground quality. (Polish Journal of Laws 2002 No.165, item 1359 of 4 October 2002), (in Polish)

[CR28] Saarela K-E, Harju L, Rajander J, Lill J-O, Heselius S-J, Lindroos A, Mattsson K (2005). Elemental analyses of pine bark and wood in an environmental study. Sci Total Environ.

[CR29] Samecka-Cymerman A, Kosior G, Kempers AJ (2006). Comparison of the moss *Pleurozium schreberi* with needles and bark of *Pine sylvestris* as biomonitors of pollution by industry in Stalowa Wola (southeast Poland). Ecotoxicol Environ Saf.

[CR30] Santamaria JM, Martin A (1997). Tree bark as a bioindicator of air pollution in Navarra, Spain. Water Air Soil Pollut.

[CR31] Sawidis T, Breuste J, Mitrovic M, Pavlovic P, Tsigaridas K (2011). Trees as bioindicator of heavy metal pollution in three European cities. Environ Pollut.

[CR32] Schulz H, Popp P, Huhn G, Stärk HJ, Schüürmann G (1999). Biomonitoring of airborne inorganic and organic pollutants by means of pine tree barks: temporal and spatial variations. Sci Total Environ.

[CR33] Serbula SM, Kalinovic TS, Ilic AA, Kalinovic JV, Steharnik MM (2013). Assessment of airborne heavy metal pollution using *Pinus* spp. and *Tilia* spp.. Aerosol Air Qual Res.

[CR34] Skwaryło- Bednarz B (2006). Total contents of selected heavy metals in forest soils of Roztocze National Park. Acta Agrophys.

[CR35] Świeboda M, Kalemba A (1979). The bark of Scots pine (*Pinus sylvestris* L.) as a biological indicator of atmospheric air pollution. Acta Soc Bot Pol.

[CR36] Szczepanowicz B, Gawroński S (2000). Water extracts from pine bark as a biological indicator of atmospheric air pollution. Sylwan.

